# Low Income Has a Negative Effect on Survival Following Diagnosis of Metastatic Colorectal Cancer—A Population‐Based Cohort Study

**DOI:** 10.1002/cam4.71357

**Published:** 2025-11-07

**Authors:** Malin Ljunggren, Caroline E. Dietrich, Cecilia Merk, Gabriella Palmer, Anna Martling, Caroline Nordenvall

**Affiliations:** ^1^ Department of Molecular Medicine and Surgery Karolinska Institutet Stockholm Sweden; ^2^ Medical Unit of Trauma, Emergency Surgery and Orthopaedics Karolinska University Hospital Stockholm Sweden; ^3^ Clinical Epidemiology Division, Department of Medicine Karolinska Institutet Stockholm Sweden; ^4^ Department of Upper Abdominal Diseases Karolinska University Hospital Stockholm Sweden; ^5^ Department of Pelvic Cancer, GI Oncology and Colorectal Surgery Unit Karolinska University Hospital Stockholm Sweden

**Keywords:** colorectal cancer, metastasis, socioeconomic factors, survival

## Abstract

**Background:**

Treatment for metastatic colorectal cancer (mCRC) has seen great advances but may not be equally available for all patients.

**Aim:**

To evaluate the impact of socioeconomic status on cancer‐specific survival after diagnosis of mCRC, with emphasis on potential temporal trends in the effect of income.

**Methods:**

This population‐based cohort study, based on 90,620 patients diagnosed with colorectal cancer in Sweden during 2007–2021 and registered in CRCBaSe, identified 33,498 patients with mCRC through 2022. We used relative survival to estimate excess mortality rate ratios (EMRRs) with 95% confidence intervals (CIs) comparing net survival by income quartiles, adjusting for sex, age, calendar year, education, marital status and birth country.

**Results:**

One‐year relative survival improved from 55% in 2007–2012 to 63% in 2017–2022. In the first years after the diagnosis of metastases there was an income gradient with the biggest contrast between high‐income and low‐income patients (EMRR (95% CI) at one year: 0.84 (0.81–0.88)). Year of mCRC diagnosis did not alter the effect of income on survival.

**Conclusions:**

Despite significant improvements in cancer‐specific survival for mCRC over the last decades, socioeconomic disparities, particularly based on income, continue to affect survival outcomes. The impact of income remains consistent from 2007 to 2021.

**Abbreviations**
CCI: Charlson comorbidity index
CI: confidence interval
cM1: clinically diagnosed metastases
cN: clinical nodal stage
CRC: colorectal cancer
CRCBaSe: CRC database
cT: clinical tumour stage
df: degrees of freedom
EMRR: excess mortality rate ratio
ICD: international classification of diseases
IPR: in‐patient registry
mCRC: metastatic CRC
OPR: out‐patient registry
pM1: pathologically diagnosed metastases
Q: quartile
SCRCR: Swedish Colorectal Cancer Registry

## 1 Introduction

Colorectal cancer (CRC) is the third most common cancer in the world [1] and metastatic disease affects 30%–40% of patients [2, 3]. During the last decade there have been great advances in the treatment of metastatic disease resulting in higher use of both metastatic surgery [4] and combination chemotherapy, targeted therapies, and immunotherapy [5]. Socioeconomic factors, including income, are associated with stage at diagnosis [6, 7], metastatic surgery [8], follow‐up after surgery, and survival after CRC [7, 9, 10, 11]. The treatments for metastatic CRC (mCRC) have become more effective which may lead to increasing differences in survival if these treatments are not equally available. On the other hand, national guidelines and structured follow‐up programs have been implemented in Sweden over the last decade, which could decrease differences in survival between socioeconomic groups. It remains unclear whether the associations between socioeconomic factors and the treatments provided, which may influence prognosis, persist.

We aimed to evaluate the impact of socioeconomic position on relative survival after diagnosis of mCRC and investigate if the effect of income on survival was affected by diagnosis year.

## 2 Materials and Methods

### 2.1 Criteria

This was a population‐based cohort study based on the previously described CRC database (CRCBaSe) linkage of the Swedish Colorectal Cancer Registry (SCRCR) to several other registries held by Statistics Sweden [12]. We included all patients diagnosed with CRC registered in SCRCR from 2007 to 2021. Exclusion criteria were age < 18 years at CRC diagnosis, previous CRC, and missing data on disposable income, education, marital status, or birth country. Patients who had clinically or pathologically diagnosed metastases (cM1/pM1) at the time of CRC diagnosis were identified as patients with synchronous metastases. Patients with synchronous metastases but no defined metastatic location in either SCRCR or in the in‐patient registry (IPR) or out‐patient registry (OPR) (international classification of diseases (ICD)‐10 codes C77, C78, C79) three months before to nine months after CRC diagnosis were excluded. Patients without synchronous metastases that did not undergo primary tumour resection (including endoscopic polypectomy) or did not enter a watch‐and‐wait program were excluded. Patients who developed a recurrence within 180 days after diagnosis of the primary CRC were transferred from the metachronous metastasis cohort to the synchronous metastasis cohort, six months being one of the two most common time points distinguishing synchronous and metachronous metastases [13, 14, 15]. Due to availability of data, the SCRCR was only used to extract metastases in 2016–2021, while the IPR/OPR were used in 2007–2022. Patients who did not develop a recurrence were excluded.

### 2.2 Definitions

Our primary exposure was equivalised disposable income, i.e., per household divided by the number of household members converted into equalised adults. This was further divided into four income quartiles (Qs) per calendar year and 5‐year age group at metastasis diagnosis. Secondary exposures were education (< 9 years, ≥ 9–12 years, ≥ 12 years), marital status (unmarried, (currently) married, divorced, widowed) and birth country (Sweden, (other) Nordic countries, (other) European countries, outside Europe). The pre‐defined confounders for *income* were age (continuous), sex (legal: male/female) and the secondary exposures. For *marital status* the confounders were the same, adding income. For *education*, confounders were age, sex and birth country. For *birth country* confounders were age, sex and marital status. The pre‐defined potential mediators for all exposures were Charlson comorbidity index (CCI) (0, 1, ≥ 2), primary location (rectal/left colon/right colon), primary clinical tumour (cT) stage (0–4) and nodal (cN) stage (0–2). Diagnosis year was included in the models to adjust for differences over time and investigated as an effect modifier in a separate model.

### 2.3 Oncological Results

Oncological data was retrieved from the SCRCR. We only used data with a date tag. Data was restricted to years 2011–2021 for coverage of a minimum of 30% per year. The number of palliative lines was based on the number of unique start dates of palliative chemotherapy lines. Data on mutational status and mismatch repair status was added to the registry in 2017.

### 2.4 Statistical Methods

Our primary outcome was net survival estimated in a relative survival framework. Expected rates stratified on calendar year, age, and sex were retrieved from the human mortality database [16]. The start of follow‐up was the date of CRC diagnosis for synchronous metastases and the date of recurrence for metachronous metastases. Follow‐up ended on the date of death, emigration, or 31 December 2022, whichever occurred first. Unadjusted cumulative relative survival was estimated using the Pohar Perme method. Flexible parametric relative survival models, with 5 degrees of freedom (df) for the baseline excess hazard rate, adjusted for confounders (as described above) were used to estimate excess mortality rate ratios (EMRRs) with 95% confidence intervals (CIs). The best fit of the models, in terms of the number of df for the baseline spline was evaluated using the Akaike and Bayesian information criteria. In a separate analysis, mediators (as described above) were added to the models to estimate the direct effect of the exposure variables. An interaction term between income and year was included in a separate confounder‐adjusted model to allow the effect of income to vary over calendar year. The significance of the interaction term was tested using a likelihood ratio test. We concluded non‐proportional excess hazards, tested using the likelihood ratio test, and therefore relaxed the proportional excess hazard assumptions throughout. We present EMRRs graphically over the first five years, together with point estimates and 95% CIs at 1‐, 3‐ and 5 years after metastasis diagnosis. Missing data were handled using complete case. Overall survival using Kaplan–Meier estimates was described for the whole cohort. Frequencies and proportions in patients and treatment characteristics were presented. Oncological results were presented and differences compared using chi‐squared tests. The significance level was 5%. Statistical analyses were done using Stata version 18 (StataCorp. 2023. Stata Statistical Software: Release 18. College Station, TX: StataCorp LLC).

## 3 Results

**FIGURE 1:**
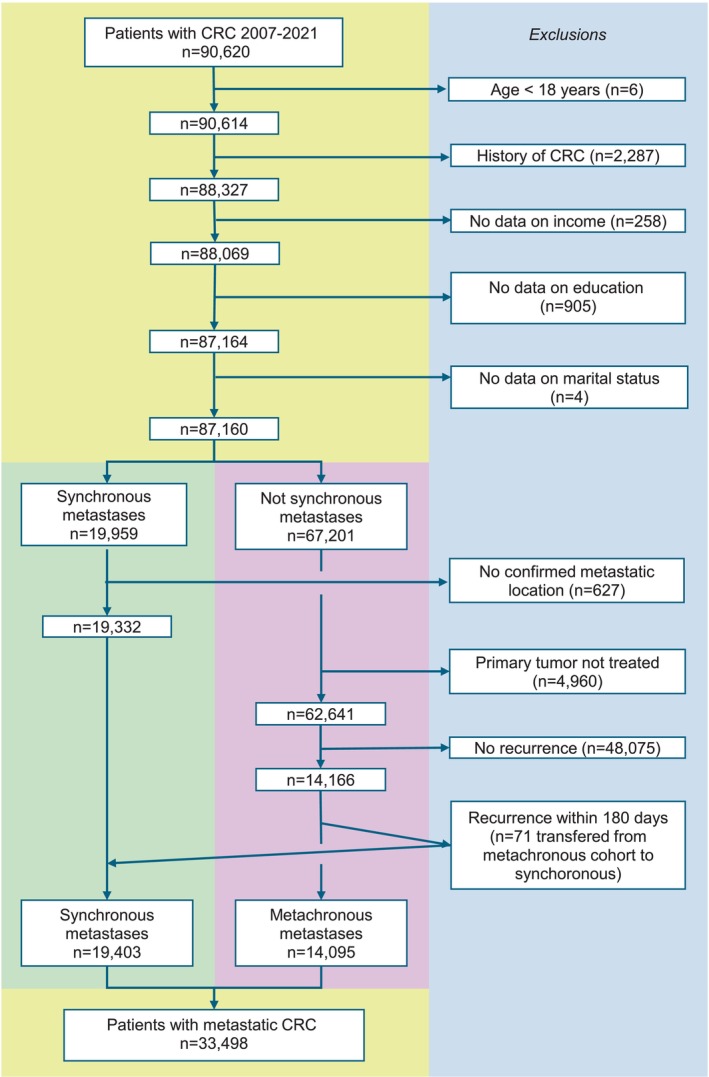
Flowchart of inclusion and exclusion criteria for the patient cohort. CRC, colorectal cancer.

In the cohort, 87,160 patients were 18 years or older, had first‐time CRC and available data on all exposures. Of them, 22% had synchronous metastases and 21% of the remaining developed metachronous metastases. This yielded a cohort of 33,498 patients with mCRC (Figure 1).

### 3.1 Patient Characteristics

The patient characteristics stratified by income Q are presented in Table 1. There were no big differences in comorbidities between the different income Qs, measured by CCI. Patients with low incomes had slightly higher cT stage, more often unknown cN stage (X), and were overrepresented in the group with right‐sided CRC.

**TABLE 1:** Patient characteristics of 33,498 patients with metastatic colorectal cancer, by income quartile from lowest (Q1) to highest (Q4).

Income quartile (Q)	Q1	Q2	Q3	Q4	All
	*N* = 8450	*N* = 8355	*N* = 8381	*N* = 8312	*N* = 33,498
Sex					
Male	3841 (45.5)	4380 (52.4)	5008 (59.8)	5221 (62.8)	18,450 (55.1)
Female	4609 (54.5)	3975 (47.6)	3373 (40.3)	3091 (37.2)	15,048 (44.9)
Age at metastasis					
Mean (SD)	70.9 (12.0)	70.9 (11.9)	70.9 (11.6)	70.8 (11.6)	70.9 (11.8)
CCI					
0	5095 (60.3)	5128 (61.4)	5124 (61.1)	5123 (61.6)	20,470 (61.1)
1	978 (11.6)	832 (10.0)	833 (9.9)	767 (9.2)	3410 (10.2)
≥ 2	2377 (28.1)	2395 (28.7)	2424 (28.9)	2422 (29.1)	9618 (28.7)
Timing of metastases					
Synchronous	5015 (59.4)	4907 (58.7)	4743 (56.6)	4738 (57.0)	19,403 (57.9)
Metachronous	3435 (40.7)	3448 (41.3)	3638 (43.4)	3574 (43.0)	14,095 (42.1)
Primary location					
Right colon	3185 (37.7)	3057 (36.6)	3010 (35.9)	3014 (36.3)	12,266 (36.6)
Left colon	2448 (29.0)	2451 (29.3)	2578 (30.8)	2606 (31.4)	10,083 (30.1)
Rectum	2755 (32.6)	2777 (33.2)	2744 (32.7)	2637 (31.7)	10,913 (32.6)
Missing	62 (0.7)	70 (0.8)	49 (0.6)	55 (0.7)	236 (0.7)
Primary CRC cT					
1–2	792 (9.4)	823 (9.9)	866 (10.3)	962 (11.6)	3443 (10.3)
3	2734 (32.4)	2895 (34.7)	2989 (35.7)	3062 (36.8)	11,680 (34.9)
4	2295 (27.2)	2119 (25.4)	2096 (25.0)	1970 (23.7)	8480 (25.3)
X	1675 (19.8)	1596 (19.1)	1543 (18.4)	1560 (18.8)	6374 (19.0)
Missing	954 (11.3)	922 (11.0)	887 (10.6)	758 (9.1)	3521 (10.5)
Primary cN					
0	2193 (26.0)	2237 (26.8)	2410 (28.8)	2280 (27.4)	9120 (27.2)
1–2	4081 (48.3)	4091 (49.0)	4065 (48.5)	4159 (50.0)	16,396 (49.0)
X	1792 (21.1)	1675 (20.0)	1570 (18.7)	1564 (18.8)	6601 (19.7)
Missing	383 (4.5)	352 (4.2)	336 (4.0)	309 (3.7)	1381 (4.1)
Marital status					
Unmarried	1896 (22.4)	1261 (15.1)	798 (9.5)	626 (7.5)	4581 (13.7)
Married	2194 (26.0)	4053 (48.5)	5579 (66.6)	5909 (71.1)	17,735 (52.9)
Divorced	2401 (28.4)	1559 (18.7)	921 (11.0)	754 (9.1)	5635 (16.8)
Widowed	1959 (23.2)	1482 (17.7)	1083 (12.9)	1023 (12.3)	5547 (16.6)
Education					
≤ 9 years	4023 (47.6)	3429 (41.0)	2641 (31.5)	1585 (19.1)	11,678 (34.9)
9–12 years	3418 (40.5)	3545 (42.4)	3671 (43.8)	3101 (37.3)	13,735 (41.0)
≥ 12 years	1009 (11.9)	1381 (16.5)	2069 (24.7)	3626 (43.6)	8085 (24.1)
Birth country					
Sweden	6806 (80.5)	7367 (88.2)	7553 (90.1)	7665 (92.2)	29,391 (87.7)
Nordic countries	535 (6.3)	407 (4.9)	360 (4.3)	262 (3.2)	1564 (4.7)
Europe	612 (7.2)	401 (4.8)	342 (4.1)	290 (3.5)	1645 (4.9)
Outside Europe	497 (5.9)	180 (2.2)	126 (1.5)	95 (1.1)	898 (2.7)

*Note:* Percentages within parentheses if nothing else is stated.

Abbreviations: CCI, Charlson comorbidity index; cN, TNM classification indicating clinical nodal status; CRC, colorectal cancer; cT, TNM classification indicating clinical T stage; Income Q, income quartile; SD, standard deviation.

### 3.2 Oncological Data

Among patients with synchronous and metachronous metastases, the presence and date of an oncological consultation were registered in 57% and 32% of patients respectively, in the years 2011–2021. Patients with high incomes more often received an oncological consultation, and at that visit they were assessed to have a better performance status. Furthermore, high‐income patients underwent more thorough diagnostic evaluations including genetic testing of the tumour and received oncological treatments more frequently (Table S1).

### 3.3 Unadjusted Survival Estimates

**FIGURE 2:**
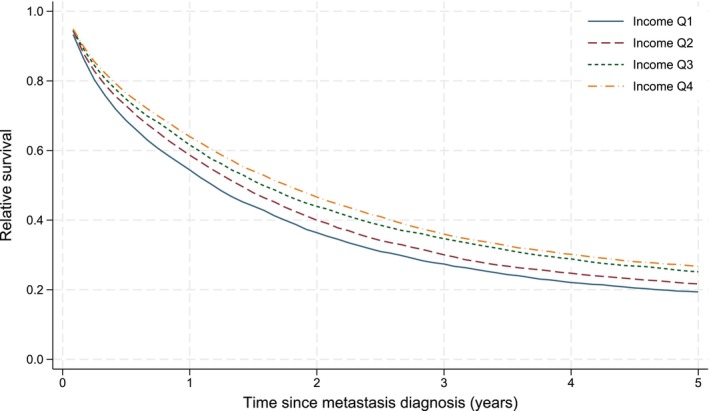
Relative (net) survival after diagnosis of metastatic colorectal cancer among 33,498 patients, by income quartiles (ranging from Q1 (lowest) to Q4 (highest)). Estimated non‐parametrically, expected survival calculated using the Pohar Perme method.

The 1‐year and 5‐year relative survival for mCRC patients was 60% and 23% respectively. The overall survival estimates were, as expected, slightly lower with a 5‐year overall survival of 20% (Figure S1). The unadjusted relative survival curves (Figure 2) show that patients with higher incomes had better relative survival than patients with lower incomes, with a stepwise gradient for each stepwise increase in income Q. The corresponding pattern was seen for education. For marital status, the best unadjusted relative survival was seen for married patients and the worst for widowed patients. For birth country, patients born in Sweden had inferior relative survival compared with patients born in non‐Nordic European and non‐European countries (Figure S2).

### 3.4 Multivariable Relative Survival Models

**FIGURE 3:**
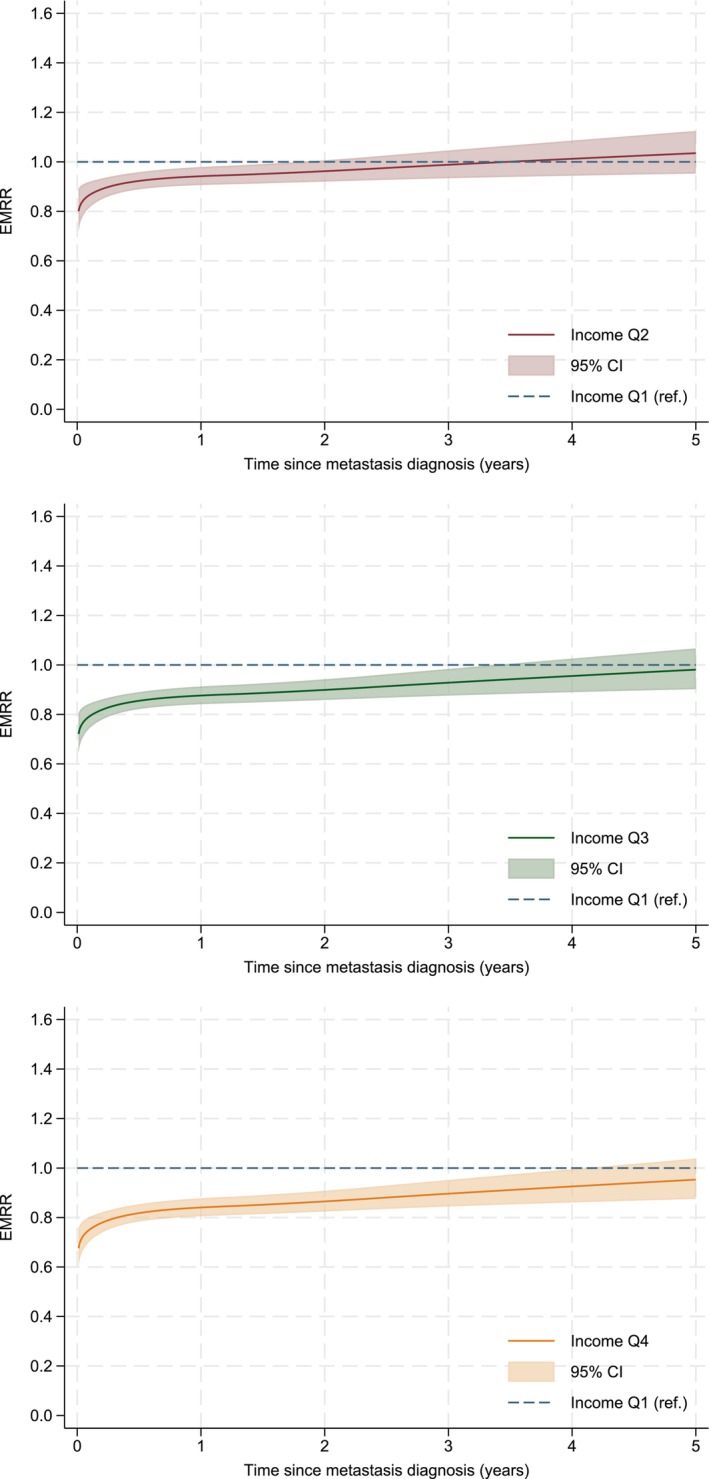
Excess mortality rate ratios (EMRR) with 95% confidence intervals (CIs) comparing cancer‐specific mortality between patients with metastatic colorectal cancer in different income quartiles (reference Q1, lowest). Estimated using a flexible parametric relative survival model allowing for non‐proportional excess hazards, and adjusted for sex, age and calendar year of diagnosis, educational status, marital status and birth country.

The confounder‐adjusted model shows that patients with higher incomes had lower excess mortality rates during the first years after metastasis diagnosis compared with low‐income patients (Figure 3). There was an income gradient where the one‐year EMRRs (95% CIs) for Q2, Q3, and Q4 compared with Q1 were 0.94 (0.91–0.98), 0.88 (0.84–0.91), and 0.84 (0.81–0.88), respectively (Table 2). The excess mortality rates were still significantly decreased for Q4 and Q3 compared with Q1 at three years after metastasis diagnosis but there was no difference at five years. Patients with higher educational levels had decreased excess mortality rates. Compared with married patients, widowed patients, unmarried patients and divorced patients, all had higher excess mortality rates in the years after metastasis diagnosis, but for variable lengths of time. Patients born in non‐Nordic European countries had lower mortality rates compared with Sweden‐born patients (Table 2, Figure S3). For all examined socioeconomic variables, inclusion of mediators in the models did not change the significance of the results (data not shown).

**TABLE 2:** 1‐, 3‐, and 5‐year point estimates of excess mortality rate ratios (EMRRs) with 95% confidence intervals (CIs) after diagnosis of metastatic colorectal cancer, by income, educational level, marital status, and birth country.

Variable	EMRR (95% CI)		
	1 year	3 years	5 years
Income^a^			
Q1	1.00	1.00	1.00
Q2	0.94 (0.91–0.98)	0.99 (0.93–1.05)	1.04 (0.95–1.12)
Q3	0.88 (0.84–0.91)	0.93 (0.88–0.98)	0.98 (0.90–1.07)
Q4	0.84 (0.81–0.88)	0.90 (0.84–0.95)	0.95 (0.87–1.04)
Education^b^			
≤ 9 years	1.00	1.00	1.00
> 9 and ≤ 12 years	0.97 (0.92–1.01)	0.96 (0.92–1.01)	0.95 (0.86–1.04)
> 12 years	0.90 (0.85–0.94)	0.91 (0.86–0.97)	0.92 (0.83–1.02)
Marital status^c^			
Married	1.00	1.00	1.00
Unmarried	1.16 (1.11–1.21)	1.15 (1.08–1.22)	1.14 (1.04–1.24)
Divorced	1.08 (1.03–1.12)	1.00 (0.95–1.06)	0.94 (0.86–1.02)
Widowed	0.96 (0.92–1.00)	0.81 (0.76–0.87)	0.69 (0.62–0.76)
Birth country^d^			
Sweden	1.00	1.00	1.00
Nordic	1.03 (0.96–1.11)	1.00 (0.91–1.11)	0.97 (0.82–1.16)
European	0.91 (0.84–0.98)	0.96 (0.87–1.06)	1.01 (0.86–1.18)
Non‐European	0.99 (0.89–1.10)	1.00 (0.88–1.14)	0.99 (0.79–1.25)

*Note:* Selection of adjustment variables was done a priori and evaluated separately for each of the listed demographical factors.

^a^ Estimated using a flexible parametric relative survival model allowing for non‐proportional excess hazards, and adjusted for sex, age and year of diagnosis, educational level, marital status, and birth country.

^b^ Estimated using a flexible parametric relative survival model allowing for non‐proportional excess hazards, and adjusted for sex, age and year of diagnosis, and birth country.

^c^ Estimated using a flexible parametric relative survival model allowing for non‐proportional excess hazards, and adjusted for sex, age and year of diagnosis, income, educational level, and birth country.

^d^ Estimated using a flexible parametric relative survival model allowing for non‐proportional excess hazards, and adjusted for sex, age and year of diagnosis, and marital status.

### 3.5 Temporal Changes

**FIGURE 4:**
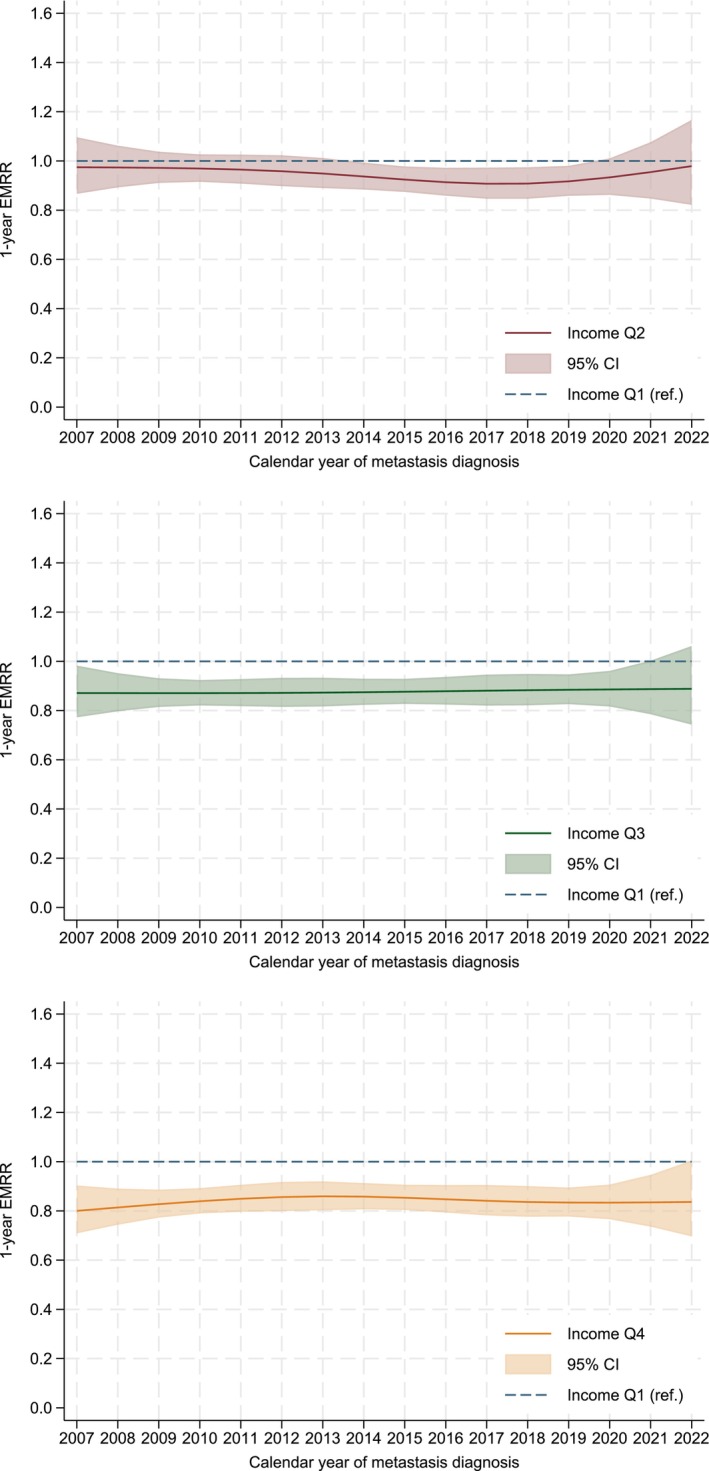
One‐year excess mortality rate ratios (EMRR) over calendar year of diagnosis, by income quartile (Q) 2, 3 and 4 compared with Q1 (lowest income). Estimated using a flexible parametric relative survival model allowing for non‐proportional excess hazards, adjusted for sex, age and calendar year of diagnosis, educational status, marital status and birth country, and including an interaction term between income and calendar year.

The one‐year relative survival increased from 55% in 2007–2011 to 63% in 2017–2022 (data not shown). There was no indication of effect modification of calendar year (*p* = 0.84 from likelihood ratio test). Figure 4 shows the estimated 1‐year EMRRs with 95% CIs from this model, illustrating the lack of temporal trend in the effect on income.

## 4 Discussion

This nationwide cohort study of 33,000 patients diagnosed with mCRC in Sweden demonstrated that income is associated with cancer‐specific mortality. Interestingly, there was no indication that the impact of income was changing over the 16‐year period. The risk reduction of cancer death for high‐income patients was highest in the first three years after metastasis diagnosis (16% lower excess mortality rates at 1 year, 10% at 3 years after metastasis diagnosis). Most deaths occur within the first three years, which is also when the impact of different treatment plans is most likely to appear. If the most effective treatments are not equally distributed between groups, a difference in cancer‐specific mortality rate in the first years after metastasis diagnosis would be expected, as was observed in this study. Our results indicate that there are no significant differences in excess mortality after three years, when the effect of initial treatments of metastases has attenuated. One‐year relative survival increased from 55% to 63% during the study period.

### 4.1 Previous Research

Socioeconomic factors have been repeatedly associated with CRC incidence, treatment and prognosis [6, 7, 8, 9, 10, 17, 18, 19, 20]. This study adds to a growing number of studies showing that socioeconomic factors are associated with CRC survival in tax‐funded universal healthcare systems [10, 21, 22]. Given the recent advances in the treatment of metastatic disease, we hypothesised that all treatments were not equally available and consequently, that mortality differences based on income had increased in recent years. Conversely, we found no change in the impact income had on survival over time. Studies with Nordic population‐based data from the 20th century have found evidence for both stable [21] and increasing [22] mortality disparities leading up to this century. Moreover, a study from New Zealand showed that the effects of income on mortality for female CRC patients had increased over time [9]. It is possible that the implementation of national guidelines and mandatory multidisciplinary team conferences has helped counteract what could have otherwise been increasing mortality disparities based on income. A previous study on non‐metastasised CRC showed decreasing, albeit not eliminated, treatment differences based on socioeconomic factors after full‐scale implementation of multidisciplinary team conferences in Sweden [23]. In summary, our study does not support that mCRC mortality disparities based on socioeconomic status are decreasing in Sweden.

### 4.2 Potential Mechanisms Relating to Patient Characteristics

Sweden's tax‐funded healthcare system is based on egalitarian principles, aiming for equal access and treatment based on need. However, differences in outcomes between socioeconomic groups may be motivated by differences in patient characteristics. High‐income patients had a better performance status (33% in Q4 vs. 22% in Q1 with World Health Organisation performance status 0) and were more likely to receive oncological treatments, including a greater number of chemotherapy lines, more combination chemotherapy, and increased use of targeted therapies. Though biomarker data did not support higher targeted therapy use in high‐income patients, the data had high missingness. Small differences in tumour characteristics were also found. Metachronous metastases, a positive prognostic factor [3, 24], were more common in high‐income patients. Differences in primary tumour location, a prognostic factor in mCRC [25], partly reflected sex distribution, as right‐sided colon cancer is more common in women [26], and women were more commonly part of the low‐income group. The proportion of rectal cancer patients in income Q1 was not higher than in other income groups, contrary to expectations based on previous research linking length of education to rectal cancer stage at diagnosis [27]. However, men were underrepresented in income Q1 and are overrepresented among patients with rectal cancer in mCRC [26], indicating that rectal cancer may be more common among low‐income patients after adjustment for patient sex. Primary tumour location was considered a potential mediator and adjusted for in a separate model. Despite adjusting for tumour location and sex, socioeconomic disparities in survival persisted, suggesting income impacts mCRC survival independently of these factors.

### 4.3 Potential Mechanisms Relating to Healthcare Access and Utilisation Patterns

Further explanations for the observed association between income and mCRC survival are that care‐seeking behaviours and attitudes towards treatment may vary by socioeconomic status. Examples include that lower socioeconomic status has been linked to lower screening participation [28], and lower theoretical acceptance of adverse effects relating to CRC chemotherapy [29, 30]. Furthermore, practical barriers to seeking healthcare may be more common among low‐income patients. Low‐income patients are likely overrepresented in rural areas, where travel times to hospitals are longer. Moreover, complimentary private health insurances increased in Sweden during the study period, although a relatively low proportion was covered (3–800,000 of 10 million), and mainly persons in working age [31]. This group may have faster access to specialist care, but treatment after CRC diagnosis should not be affected. Thus, the effect of private health insurance on our results is likely limited. Lastly, participation in clinical trials may differ by socioeconomic status. Patients living in areas with higher socioeconomic levels more often participate in clinical trials [32] and clinical trials are mostly run from large academic centres in urban areas [33]. We have previously demonstrated that patients managed in university hospitals in Sweden undergo metastatic surgery to a higher extent and have superior survival compared to patients managed in non‐university hospitals [4], which could be related to clinical trial participation rates. Implementation of CRC screening is underway in Sweden, which carries the potential to decrease some of these barriers to mCRC care among patients with lower income. An area of future research could be to examine if screening decreases income‐related differences in mCRC survival.

### 4.4 Education, Marital Status and Birth‐Country

High education level was associated with a lower excess mortality rate at 1 year after metastasis diagnosis. High educational level was more common in high‐income patients and the mechanisms that underlie the associations with survival are likely similar for income and education. Married patients had lower excess mortality rates during the first year after diagnosis of metastatic disease, compared with unmarried, divorced and widowed patients. Most patients, but especially married patients, involve their family members in treatment decisions after newly diagnosed CRC or lung cancer [34], and married patients and patients living with a partner have better survival outcomes from CRC [10, 35]. Widowed patients had higher excess mortality during the first year after mCRC diagnosis, but survivors had a lower excess mortality rate than married patients thereafter. The lower mortality rates among patients alive after this initial period, may reflect a healthier subset of widowed patients, despite the high early mortality and poor prognosis in this group. Although speculative, this indicates that there is potential to improve survival in this subgroup. Size of social networks and perceived social support are among factors associated with cancer survival [36], that could also be targeted in societal interventions aiming to improve health in this patient group. In the multivariable model, only patients born in non‐Nordic European countries compared with patients born in Sweden had significantly lower excess mortality rates at 1 year after mCRC diagnosis, despite higher unadjusted cumulative relative survival for patients born in non‐Nordic European countries and non‐European countries in the first five years of follow up. This was somewhat unexpected because only a few potential confounders were adjusted for. These results could be influenced by a type of misclassification bias if the expected survival for non‐European born patients is not comparable with the Swedish standard population used to calculate relative survival. There is some support for a healthy migrant effect, especially regarding mortality, in Sweden, i.e., that migrants are healthier than their new country peers [37]. If the expected survival for non‐European born patients is underestimated due to higher all‐cause mortality in the standard population, this could lead to overestimated cancer‐specific survival for non‐Nordic born patients and a falsely reduced EMRR (closer to one). This could be an interesting area for further research.

### 4.5 Strengths and Weaknesses

This is a large‐scale population‐based study with data derived from high‐quality national registries which makes the results reliable and robust. The study covers a recent 16‐year long period allowing for longitudinal analysis of trends over time. Moreover, the use of relative survival, allows for analyses on how the cancer has affected prognosis. The oncological treatment data was compromised by missing data but was not included in any models. The generalisability of our results is limited to other populations with similar wealth distributions and healthcare systems. While we tried to account for confounders by adjusting in multivariable analyses, there may be unaccounted variables left, i.e., residual confounding.

## 5 Conclusions

Patients with higher disposable incomes, who are married and have higher educations have superior cancer‐specific survival after diagnosis of mCRC. Survival increased during the 16‐year study period, but the effect income had on survival of mCRC did not change over time. Efforts to decrease differences in survival based on socioeconomic status need to encompass larger preventative measures including health promotion and cancer symptom awareness.

## Author Contributions

**Malin Ljunggren:** conceptualization, methodology, formal analysis, writing – original draft, data curation, visualization. **Caroline E. Dietrich:** conceptualization, methodology, data curation, writing – review and editing, validation, visualization. **Cecilia Merk:** conceptualization, writing – review and editing. **Gabriella Palmer:** conceptualization, writing – review and editing, supervision. **Anna Martling:** conceptualization, writing – review and editing, resources, funding acquisition. **Caroline Nordenvall:** conceptualization, methodology, writing – review and editing, resources, supervision, funding acquisition.

## Ethics Statement

Ethical permissions (DNR: 2014/71‐31/1, 2018/328‐32, 2021‐00342, 2023‐03305‐02) were given by the Regional Board of the Ethical Committee in Stockholm, Sweden.

## Consent

In accordance with the ethical permission, the need to obtain informed consent was waived.

## Conflicts of Interest

The authors declare no conflicts of interest.

## Supporting information


**Figure S1:** Overall survival for 33,498 patients diagnosed with metastatic colorectal cancer estimated using the Kaplan–Meier method.

**Figure S2:** Relative survival after diagnosis of metastatic colorectal cancer among 33,498 patients, by educational status, marital status, and birth country. Estimated non‐parametrically, expected survival calculated using the Pohar Perme method.

**Figure S3:** Excess mortality rate ratios (EMRR) with 95% confidence intervals (CIs) comparing cancer‐specific mortality between patients with metastatic colorectal cancer, by educational status^a^, marital status^b^, and birth country^c^. Selection of adjustment variables was done a priori, and evaluated separately for each of the listed demographical factors. ^a^Estimated using a flexible parametric relative survival model allowing for non‐proportional excess hazards, and adjusted for sex, age and year of diagnosis, and birth country. ^b^Estimated using a flexible parametric relative survival model allowing for non‐proportional excess hazards, and adjusted for sex, age and year of diagnosis, income, educational level, and birth country. ^c^Estimated using a flexible parametric relative survival model allowing for non‐proportional excess hazards, and adjusted for sex, age and year of diagnosis, and marital status.

**Table S1:** Oncological treatment by income quartile from 2011 to 2021 (in case oncological visit date was registered). Percentages within parenthesis if nothing else is stated.

## Data Availability

The data are available on request from the steering group of SCRCR and CRCBaSe with the appropriate approvals, but restrictions apply. More information is available from the CRCBaSe website: https://cancercentrum.se/samverkan/cancerdiagnoser/tjocktarm‐andtarm‐och‐anal/tjock‐‐och‐andtarm/kvalitetsregister/forskning/forskningsdatabas/.
