# Supplementary figures and images for: Low Income Has a Negative Effect on Survival Following Diagnosis of Metastatic Colorectal Cancer—A Population‐Based Cohort Study
Source: Cancer Med. 2025 Nov 7;14(21):e71357. doi: 10.1002/cam4.71357 (PMC12593528; doi:10.1002/cam4.71357)

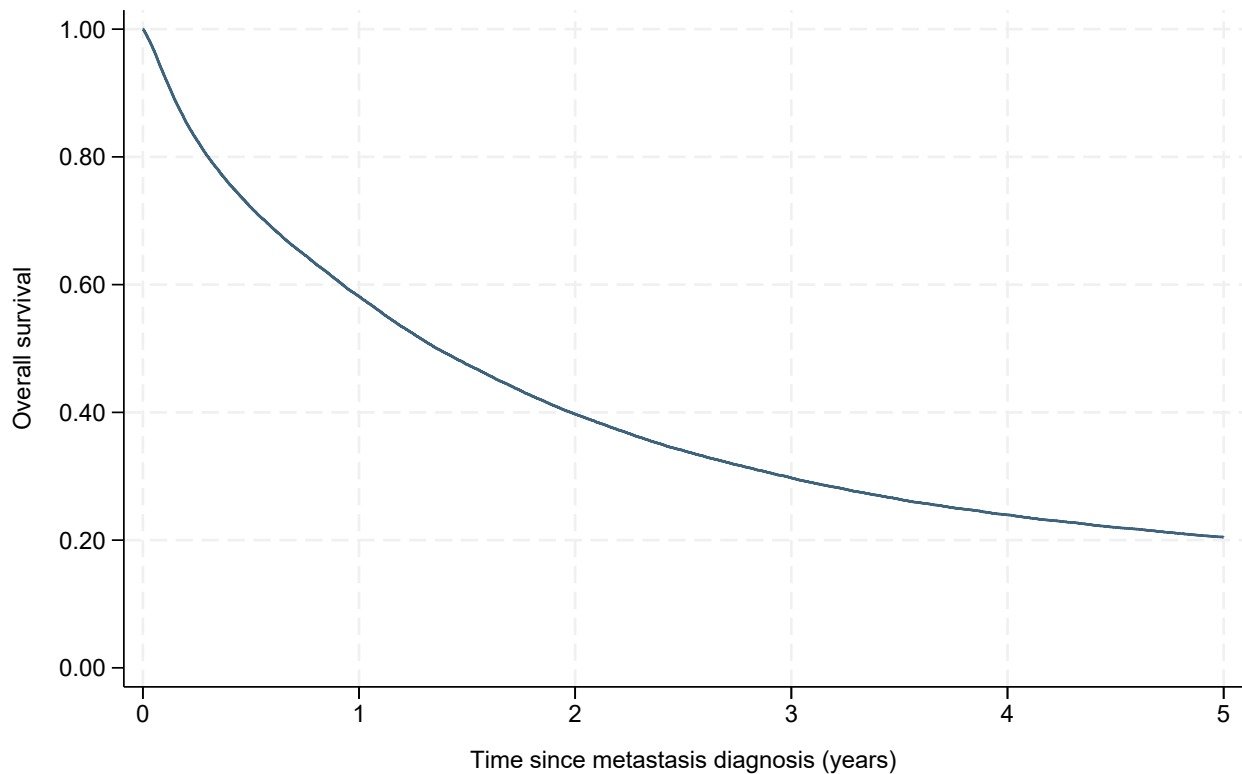

Number at risk

|              |       |       |       |      |      |      |
|--------------|-------|-------|-------|------|------|------|
| All patients | 33498 | 18624 | 11600 | 7792 | 5528 | 4135 |
|--------------|-------|-------|-------|------|------|------|

Supplement: Supplementary file 1 — Figure S1: Overall survival for 33,498 patients diagnosed with metastatic colorectal cancer estimated using the Kaplan–Meier method. [file CAM4-14-e71357-s004.pdf]

## Education

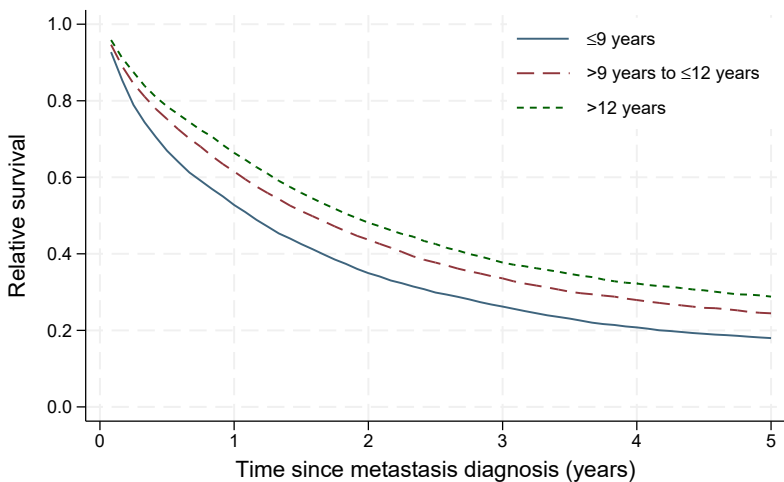

## Marital status

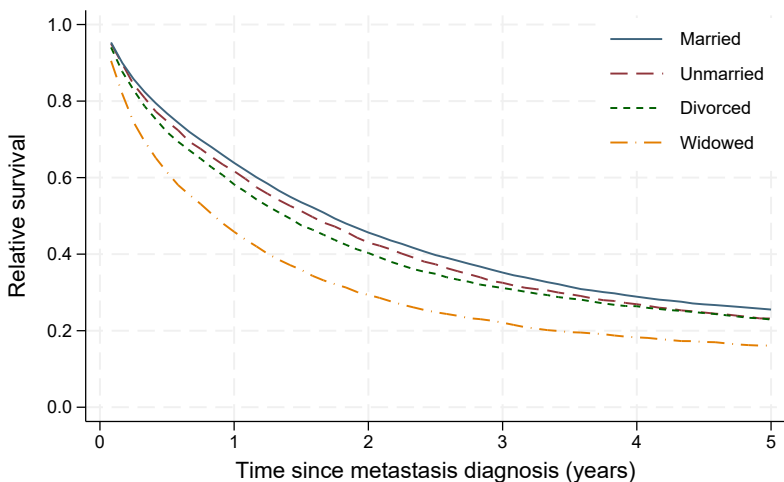

## Birth country

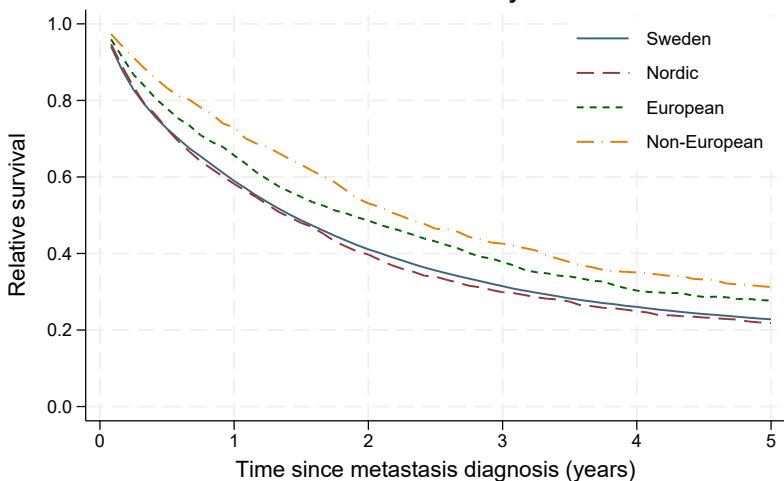

Supplement: Supplementary file 2 — Figure S2: Relative survival after diagnosis of metastatic colorectal cancer among 33,498 patients, by educational status, marital status, and birth country. Estimated non‐parametrically, expected survival calculated using the Pohar Perme method. [file CAM4-14-e71357-s002.pdf]

Education

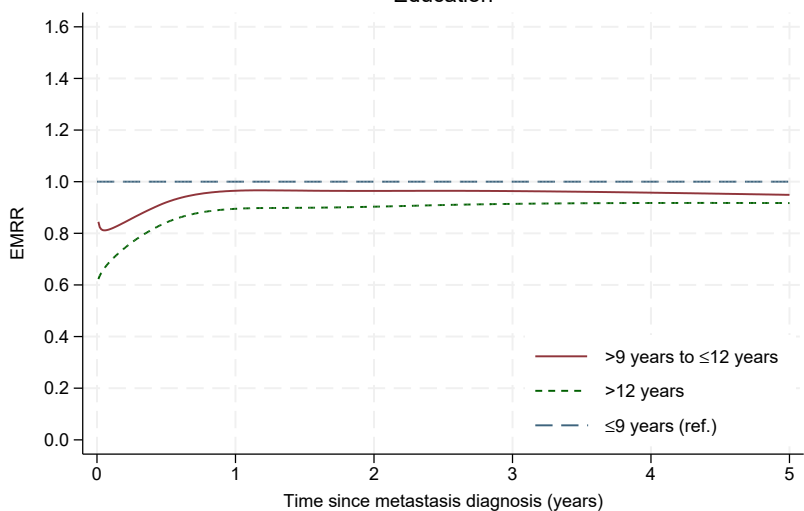

Marital status

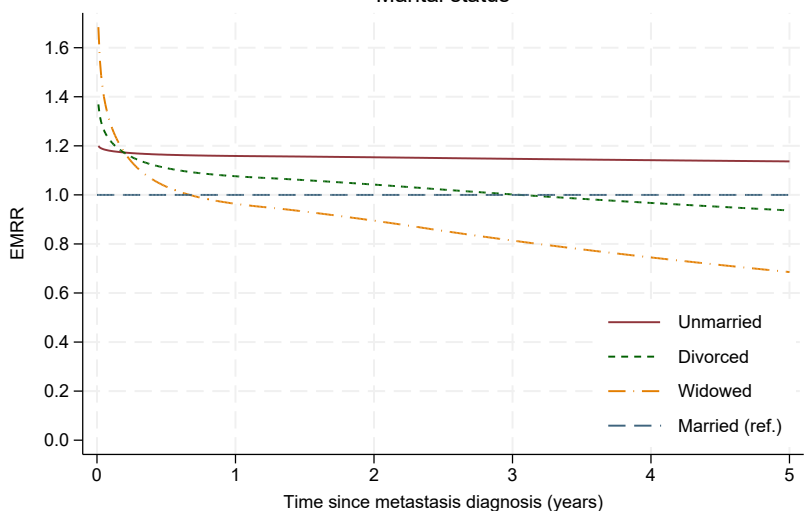

Birth country

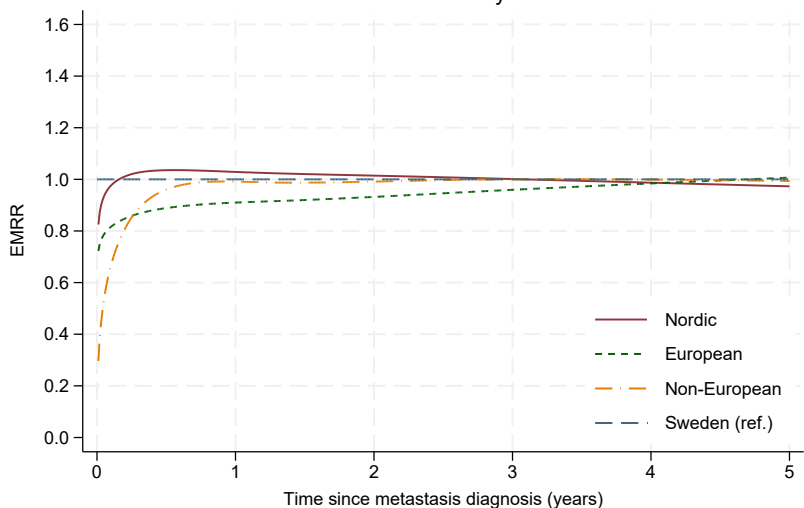

Supplement: Supplementary file 3 — Figure S3: Excess mortality rate ratios (EMRR) with 95% confidence intervals (CIs) comparing cancer‐specific mortality between patients with metastatic colorectal cancer, by educational statusa, marital statusb, and birth countryc. Selection of adjustment variables was done a priori, and evaluated separately for each of the listed demographical factors. aEstimated using a flexible parametric relative survival model allowing for non‐proportional excess hazards, and adjusted for sex, age and year of diagnosis, and birth country. bEstimated using a flexible parametric relative survival model allowing for non‐proportional excess hazards, and adjusted for sex, age and year of diagnosis, income, educational level, and birth country. cEstimated using a flexible parametric relative survival model allowing for non‐proportional excess hazards, and adjusted for sex, age and year of diagnosis, and marital status. [file CAM4-14-e71357-s001.pdf]
