# Supplementary material for: Low Income Has a Negative Effect on Survival Following Diagnosis of Metastatic Colorectal Cancer—A Population‐Based Cohort Study
Source: Cancer Med. 2025 Nov 7;14(21):e71357. doi: 10.1002/cam4.71357 (PMC12593528; doi:10.1002/cam4.71357)
Supplement: Supplementary file 4 — Table S1: Oncological treatment by income quartile from 2011 to 2021 (in case oncological visit date was registered). Percentages within parenthesis if nothing else is stated. [file CAM4-14-e71357-s003.docx]

**SUPPLEMENTARY TABLE 1** Oncological treatment by income quartile from 2011-2021 (in case oncological visit date was registered). Percentages within parenthesis if nothing else is stated.

|  | IQ1  N=2,813 | IQ2  N=2,967 | IQ3  N=3,105 | IQ4  N=3,136 | p-value |
| --- | --- | --- | --- | --- | --- |
| Oncological treatment |  |  |  |  |  |
| Yes | 2,392 (85 %) | 2,640 (89 %) | 2,786 (90 %) | 2,840 (91 %) | <0.001 |
| No | 337 (12 %) | 263 (9 %) | 249 (8 %) | 240 (8 %) |  |
| Missing | 84 (3 %) | 64 (2 %) | 70 (2 %) | 55 (2 %) |  |
| Test mutational status* |  |  |  |  |  |
| Yes/all | 489/1,252 (39 %) | 551/1,335 (41 %) | 632/1,407 (45 %) | 609/1,394 (44 %) | 0.055 |
| BRAF mutation/all tested | 68/489 (14%) | 88/551 (16%) | 105/632 (17%) | 101/609 (17%) | 0.157 |
| KRAS mutation/ all tested | 232/489 (47%) | 262/551 (48%) | 305/632 (48%) | 278/609 (46%) | 0.232 |
| NRAS mutation/ all tested | 19/489 (4%) | 31/551 (6%) | 38/632 (6%) | 32/609 (5%) | 0.219 |
| Test MMR status* |  |  |  |  |  |
| Yes/all | 209/1,252 (17 %) | 223/1,335 (17 %) | 290/1,407 (21 %) | 302/ 1,394 (22 %) | 0.003 |
| MSI high/ all tested | 17/ 209 (8%) | 26/223 (12%) | 24/290 (8%) | 20/302 (7%) | 0.613 |
| WHO performance status, lowest after diagnosis of CRC metastases |  |  |  |  |  |
| 0 | 611 (22 %) | 772 (26 %) | 896 (29 %) | 1,041 (33 %) | <0.001 |
| 1 | 651 (23 %) | 649 (22 %) | 651 (21 %) | 594 (19 %) |  |
| 2 | 252 (9 %) | 257 (9 %) | 195 (6 %) | 167 (5 %) |  |
| 3 | 87 (3 %) | 53 (2 %) | 71 (2 %) | 52 (2 %) |  |
| Missing | 1,212 (43 %) | 1,236 (42 %) | 1,292 (42 %) | 1,281 (41 %) |  |
| Number of palliative chemotherapy lines started |  |  |  |  | <0.001 |
| 0 or missing | 1,227 (44 %) | 1,207 (41 %) | 1,225 (39 %) | 1,178 (38 %) |  |
| 1 | 846 (30 %) | 835 (28 %) | 860 (28 %) | 848 (27 %) |  |
| 2 | 364 (13 %) | 458 (15 %) | 483 (16 %) | 520 (17 %) |  |
| 3 | 187 (7 %) | 22 (7 %) | 259 (8 %) | 249 (8 %) |  |
| 4 | 179 (6 %) | 245 (8 %) | 278 (9 %) | 340 (11 %) |  |
| FOLFOX/CAPOX** | 878 (31 %) | 1,019 (34 %) | 1,072 (35 %) | 1,178 (38 %) | <0.001 |
| FOLFIRI/CAPIRI** | 698 (25 %) | 843 (28 %) | 935 (30 %) | 988 (32 %) | <0.001 |
| FOLFOXIRI/CAPOXIRI** | 34 (1 %) | 43 (1 %) | 55 (2 %) | 68 (2 %) | 0.023 |
| Bevacizumab*** | 344 (12 %) | 396 (13 %) | 499 (16 %) | 573 (18 %) | <0.001 |
| Panitumumab/ cetuximab*** | 268 (10%) | 314 (11 %) | 366 (12 %) | 368 (12 %) | 0.015 |

** Restricted to period 2017-2021. P-values on occurrence of mutations and MSI-high calculated including group with missing test.*

*** Same patient could receive e.g. FOLFOX and FOLFIRI but not at the same time (different lines).*

**** At any time after diagnosis of metastasis.*
